# High Phobic Anxiety Is Related to Lower Leukocyte Telomere Length in Women

**DOI:** 10.1371/journal.pone.0040516

**Published:** 2012-07-11

**Authors:** Olivia I. Okereke, Jennifer Prescott, Jason Y. Y. Wong, Jiali Han, Kathryn M. Rexrode, Immaculata De Vivo

**Affiliations:** 1 Channing Laboratory, Department of Medicine, Brigham and Women's Hospital and Harvard Medical School, Boston, Massachusetts, United States of America; 2 Department of Epidemiology, Harvard School of Public Health, Boston, Massachusetts, United States of America; 3 Program in Molecular and Genetic Epidemiology, Harvard School of Public Health, Boston, Massachusetts, United States of America; 4 Clinical Research Program, Department of Dermatology, Brigham and Women's Hospital and Harvard Medical School, Boston, Massachusetts, United States of America; 5 Department of Psychiatry, Brigham and Women's Hospital and Harvard Medical School, Boston, Massachusetts, United States of America; 6 Division of Preventive Medicine, Department of Medicine, Brigham and Women's Hospital and Harvard Medical School, Boston, Massachusetts, United States of America; Baylor College of Medicine, United States of America

## Abstract

**Background:**

Chronic psychological distress has been linked to shorter telomeres, an indication of accelerated aging. Yet, little is known about relations of anxiety to telomeres. We examined whether a typically chronic form of anxiety – phobic anxiety – is related to telomere length.

**Methodology/Principal Findings:**

Relative telomere lengths (RTLs) in peripheral blood leukocytes were measured by quantitative real-time polymerase chain reaction among 5,243 women (aged 42–69 years) who: were participants in the Nurses' Health Study; were controls in prior case-control studies of telomeres and disease, or randomly selected healthy participants in a cognitive function sub-study; had completed the Crown-Crisp phobic index proximal to blood collection. Adjusted least-squares mean RTLs (*z*-scores) were calculated across phobic categories. Higher phobic anxiety was generally associated with lower RTLs (age-adjusted p-trend = 0.09); this association was similar after adjustment for confounders – paternal age-at-birth, smoking, body mass index (BMI) and physical activity (p-trend = 0.15). Notably, a threshold was identified. Among women with Crown-Crisp<6 points, the multivariable-adjusted least-squares mean RTL *z*-score = 0.02 standard units; however, among the most phobic women (Crown-Crisp≥6), the multivariable-adjusted least-squares mean RTL *z*-score = −0.09 standard units (mean difference = −0.10 standard units; p = 0.02). The magnitude of this difference was comparable to that for women 6 years apart in age. Finally, effect modification by BMI, smoking and paternal age was observed: associations were stronger among highly phobic women with BMI≥25 kg/m^2^, without smoking history, or born to fathers aged ≥40 years.

**Conclusions/Significance:**

In this large, cross-sectional study high phobic anxiety was associated with shorter telomeres. These results point toward prospective investigations relating anxiety to telomere length change.

## Introduction

Telomeres are repetitive DNA sequences at the ends of eukaryotic chromosomes that undergo attrition each time a somatic cell divides. Factors that accelerate attrition include oxidative stress and inflammation [1], [2]. Consequently, average telomere length (TL) reflects cumulative damage from these exposures, and is a potential indicator of biological aging [3]. Furthermore, by protecting chromosomal ends, telomeres maintain genomic stability [4], [5], [6]; critically short telomeres lead to DNA damage (e.g., end-to-end fusion, atypical recombination or rearrangement) implicated in development of several age-related diseases [7]. For example, epidemiological studies have reported associations between shorter TLs and increased risks of cancers [8], [9] and cancer mortality [10], cardiovascular disease [7], cognitive decline and dementia [11], [12]. Because telomere shortening may underlie many adverse health outcomes in aging, it is important to identify addressable risk factors.

An emerging literature implicates psychological distress and mood disorders, both highly prevalent in women, as potential paths toward accelerated aging [13], [14], [15], [16]. Prior work has indentified relations of depression to higher levels of inflammatory mediators and oxidative stress [17]. Although less is known about its relations to these mechanistic paths, anxiety could also be a risk factor for morbidity or mortality in aging. In a previous investigation in the Nurses' Health Study (NHS), phobic anxiety was significantly related to higher levels of inflammatory markers [18] and a elevated risk of sudden cardiac death and fatal coronary disease [19]. Importantly, phobic anxiety is treatable; thus, any potential impacts on telomere shortening may be amenable to prevention through early identification and treatment. However, there have been few prior investigations of anxiety and telomere shortening.

Thus, we conducted an examination of the relation of phobic anxiety to peripheral blood leukocyte TLs (LTLs) in 5,243 participants of the Nurses' Health Study, who ranged from age 42–69 years (mean = 59) when they provided self-reports of phobic symptoms and blood samples. As phobic anxiety tends to be chronic – median age at onset is 11 years [20], and course tends to be highly persistent without treatment [21], [22], [23] – we hypothesized strong inverse associations between phobic anxiety and LTLs measured at mid- and later-life.

## Methods

### Study population

The Nurses' Health Study (NHS) included 121,700 U.S., female registered nurses, aged 30 to 55 years at the study's inception in 1976. Since then, participants have completed biennial mailed questionnaires updating information on numerous lifestyle factors and health outcomes; total follow-up exceeds 90%. Details regarding the NHS and validation of various health exposures and endpoints have been published previously [24], [25], [26]. During the 1988–1990 questionnaire cycle, participants were asked to complete the Crown-Crisp Index (CCI) of phobic anxiety [27], [28]. From 1989 to 1990, 32,826 NHS participants provided blood samples; details of the blood collection and archival methods have been described previously [29], [30]. The protocols for the NHS, the NHS blood collection, and the current study were approved by the Institutional Review Board of Brigham and Women's Hospital, Boston, MA, USA. Blood donors gave written informed consent to having their samples used for research purposes. In addition, all data was analyzed anonymously.

To determine the final study population, we considered participants from nested case-control studies of pre-diagnostic LTLs and incident cancers and cardiovascular disease (CVD), and from a study of cognitive function (n = 9,190) [12], [31], [32], [33]. Although blood collections occurred among both cases and controls prior to onset of the diseases of interest, we utilized the most conservative approach and minimized possible bias by including only controls and the random sample of cognitive study participants (n = 5,415). We excluded women with insufficient CCI data (i.e., missing >2 items) (n = 146) or missing LTL values (n = 26). Thus, the sample for analysis included 5,243 women.

### Assessment of phobic anxiety: the Crown-Crisp index

The CCI measures symptoms of phobic anxiety; it has been validated in psychiatric outpatient settings and found to discriminate patients with general anxiety and phobias from healthy controls or those with other conditions (i.e., obsessive-compulsive or depressive) [27], [28]. The CCI primarily covers aspects of “fear” disorders, such as panic and agoraphobia. The index features 8 self-rated questions, and total scores range from 0 to 16 points (higher scores indicate higher anxiety). For those missing data on 1 or 2 items, we chose the more conservative approach of basing the CCI sum score on only the answered items [34] (i.e., rather than imputing scores using the mean [19], [35]). CCI scores are not normally distributed, and in keeping with other work [36], [37] we categorized CCI scores into 5 groups: 0 or 1 point (reference group); 2 points; 3 points; 4 or 5 points; and ≥6 points (highest phobic anxiety group; the range of CCI scores in this fifth group = 6–16 points). Of note, the CCI is reliable in the NHS cohort: the Cronbach coefficient alpha for scores in 1988 was 0.62 – comparable to the coefficient of 0.69 originally reported by Crown and Crisp among diagnosed patients and controls [28]; item-total correlations were also in the desirable range [38] (0.26–0.43). Furthermore, identical items were featured on the 2004 NHS questionnaire; the intraclass correlation coefficient (95% confidence interval [CI]) for the pair of CCI scores in 1988 and 2004 was 0.61 (0.61–0.62), and the Fleiss-Cohen-weighted kappa (95% CI) for consistency of the 5 phobic categories was 0.56 (0.55–0.57) – an indication that these measures can reliably represent long-term phobic anxiety levels.

### Measurement of leukocyte telomere length

Genomic DNA was extracted from peripheral blood leukocytes using the QiAmp (Qiagen) 96-spin blood protocol. Our outcome measure was relative LTL (RTL) using quantitative real-time polymerase chain reaction (Q-PCR) [8]. Average RTL was calculated as the exponentiated ratio of Telomere repeat copy number to Single gene (*36B4*) copy number (T/S) corrected for a reference sample [39]. Laboratory technicians masked to participant characteristics assayed each sample in triplicate. Quality control samples were interspersed on each plate to assess variability. In all nested case-control studies, coefficients of variation (CVs) for the telomere and single gene assay were <4%, and CVs for the exponentiated T/S ratio were <17%. Although this assay provides a relative measurement of telomere length, T/S ratios correlate well with absolute telomere lengths determined by Southern blot (*r* = 0.82, *p*<0.0001) [39]. As expected, significant correlations have been found in the NHS cohort between shorter telomeres and key predictors: age (Spearman partial rank correlation coefficient [*r_s_*] = −0.09, *p*<0.0001), smoking pack-years (*r_s_* = −0.04, p = 0.0005) and body mass index (BMI) (*r_s_* = −0.04, p = 0.0008) [40].

### Assessment of covariates

In addition to age-at-blood collection, we considered number of variables potentially associated with phobic anxiety and telomere length. Using the biennial questionnaires completed proximal (1988–1990) to blood collection and a supplemental questionnaire administered at blood collection, we ascertained factors that have been related to TLs in the NHS and elsewhere: BMI (kg/m^2^); physical activity (total metabolic equivalent hours of activity per week [MET-hrs/wk]) [40]; cigarette smoking (pack-years) [8], [41], [42]; and paternal age-at-participant's birth (15–24, 25–29, 30–34, 35–39, ≥40 years) [43]. Also, we obtained information on numerous health, social and lifestyle factors: postmenopausal hormone use, with duration (never or pre-menopausal; past; current <5 years or ≥5 years); oral contraceptive use (ever/never); anthropometric measures (e.g., waist-hip ratio, waist circumference); education (associate's, bachelor's, or master's/doctoral degree) [44]; race/ethnicity; work status (e.g., employed outside home, homemaker, retired, etc.) [45]; alcohol intake (grams/day); daily multivitamin use [46]; medication use, including benzodiazepines (BZDs) (N.B.: regular diazepam use was reported as yes/no in 1980 and 1982; data on regular antidepressant use was not available until 1996); and presence/absence of chronic comorbidities (e.g., diabetes, hypertension). Finally, using a validated semi-quantitative food frequency questionnaire [47] administered in 1990, we assessed dietary factors (e.g., calories, macronutrients, n-6 and n-3 fatty acids [48], caffeine intake, vitamin intake [46], antioxidant fruit and vegetable consumption).

### Statistical analyses

The distribution of RTLs varied across batch sets from each nested case-control study and the cognitive study. Thus, we computed RTL *z*-scores within each batch, after calculating the natural logarithm to improve normality.

To examine potential for confounding, we first examined univariate associations of the factors described above with phobic categories and RTL *z*-scores; variables with significant or borderline relations to phobic anxiety or RTL were addressed in preliminary model-building steps. We assessed for threshold associations using the 5 phobic categories and for linear trends by treating phobic category as an ordinal predictor.

To examine the association between phobic anxiety and RTLs, we estimated adjusted least-squares mean RTL *z*-scores using three generalized linear models. In the first model, we adjusted for age-at-blood draw in years. In the second model, we additionally adjusted for the largest potential contributors to confounding: BMI, physical activity, pack-years of smoking and paternal age. Sequentially, we considered other covariates (described above) as potential confounders, based on prior literature, putative associations to oxidative stress, or preliminary univariate analyses. As there were no changes in the estimates with inclusion of these covariates, they were omitted from the final multivariate model. Thus, the third model was the primary model and it included: participant age, paternal age, smoking, BMI and physical activity; indicator variables were created where covariates had missing values.

We conducted key secondary analyses to address sensitivity and to further explore phobic-telomere associations. First, we further adjusted the primary model for major conditions that are potential confounders and/or intermediates: hypertension, diabetes, dyslipidemia, and CVD. Second, we utilized multiplicative interaction terms to evaluate whether associations differed by participant age (<60/≥60 years), older parental age (mother <35/≥35 years; father <40/≥40 years [49]), BMI (18.5–25 vs. ≥25 kg/m^2^), smoking (ever/never), physical activity (above/below median), or BZD use (yes/no), as both biology and prior literature support potential modifying roles [50], [51], [52]. Third, as some participants had major comorbidities that could contribute to higher self-reported anxiety symptom levels as well as shorter telomeres (e.g., obstructive airways diseases show strong associations with panic and anxiety) [53] we conducted separate analyses excluding women diagnosed with prevalent CVD [54], diabetes [55], or chronic obstructive airways diseases (asthma, chronic bronchitis or emphysema) [26], [56] prior to or concurrent with blood collection (total n-excluded = 594). Finally, we conducted a separate analysis to explore relations of individual CCI items to RTLs, adjusting for age. All statistical analyses were performed using SAS version 9.2 (SAS Institute Inc, Cary, NC). Hypothesis testing was 2-sided, with α = 0.05.

## Results

Table 1 shows sample characteristics at blood draw, across phobic anxiety levels. Women in the highest phobic category were generally less healthy than those in the lowest category: e.g., higher phobic anxiety was associated with lower physical activity and educational attainment, and with higher BMI, smoking pack-years, and prevalence of hypertension, CVD, obstructive airways diseases, diabetes, and dyslipidemia. Women with higher phobic symptoms were less likely to use daily multivitamins or to have used oral contraceptives, and more likely to have used BZDs. Regarding diet, slightly higher daily calories, lower energy from carbohydrates, protein and omega-3 fatty acids, and lower vitamin D intake were observed among more phobic women; there were no other consistent differences by phobic anxiety in intake of other dietary factors.

**Table 1:** Characteristics by phobic anxiety categories (lowest to highest) at blood draw (n = 5,243)*.

CHARACTERISTIC	*Crown-Crisp Index Phobic Anxiety Scores*				
	*0 or 1*	*2*	*3*	*4 or 5*	*≥6*
Number of participants	1,782	1,024	816	1,022	599
Median Crown-Crisp phobic index	1.0	2.0	3.0	4.0	7.0
Mean (SD) age (years)	59.3 (6.5)	59.1 (6.6)	59.1 (6.6)	59.4 (6.5)	59.4 (6.5)
Mean (SD) telomere length (z-score)	0.01 (1.00)	0.05 (1.00)	−0.01 (1.00)	0.02 (1.01)	−0.10 (0.96)
Mean (SD) body mass index (kg/m^2^)	25.1 (4.5)	24.9 (4.5)	25.4 (4.4)	25.4 (4.8)	26.0 (5.3)
Mean (SD) alcohol intake (g/day)	5.4 (9.4)	5.6 (9.7)	5.7 (9.6)	5.6 (10.2)	5.4 (10.5)
Mean (SD) physical activity (METs/week)	17.2 (18.3)	15.9 (17.8)	15.9 (18.3)	15.3 (16.5)	13.1 (14.4)
Mean (SD) pack-years of smoking	11.5 (17.5)	13.3 (19.1)	14.0 (19.2)	13.4 (19.1)	14.6 (19.9)
Mean (SD) paternal age at participant's birth	31.6 (7.2)	31.2 (6.9)	31.4 (6.8)	31.7 (7.3)	31.0 (6.7)
Caucasian race (%)	95.5	96.5	94.9	96.2	95.3
Education (%)					
Bachelor's	22.6	20.5	17.7	18.4	17.5
Master's/doctoral	12.7	10.5	9.6	7.1	4.8
Multivitamin use (%)	40.2	40.8	40.2	38.1	35.0
Prior benzodiazepine use (%)	4.2	5.6	5.2	7.2	8.2
Ever used oral contraceptives (%)	42.7	42.9	42.2	41.6	36.6
Postmenopausal hormone use (%)					
Pre-menopause status, or never	46.0	49.2	44.9	42.6	43.9
Past	15.2	16.2	17.3	20.1	18.5
Current, <5 years	15.5	14.0	17.4	13.0	14.6
Current, ≥5 years	23.3	20.6	20.4	24.3	23.0
Asthma, chronic bronchitis, or emphysema (%)	4.7	6.1	5.3	4.9	8.5
Hypertension (%)	27.6	28.5	30.9	32.2	34.2
Diabetes (%)	3.9	4.8	3.6	5.1	6.0
Heart disease (%)	1.7	2.1	1.8	2.6	3.8
Elevated cholesterol (%)	40.2	42.4	43.3	46.5	46.9
Nutrient intakes, in means (SDs)					
Total energy (kcal/day)	1746.2 (496.5)	1739.2 (465.7)	1765.4 (485.3)	1774.2 (472.2)	1793.7 (513.1)
Carbohydrate (% of energy)	50.8 (18.9)	50.0 (17.9)	49.4 (17.4)	49.0 (17.0)	48.8 (17.1)
Protein (% of energy)	19.1 (6.7)	18.9 (6.4)	18.6 (6.8)	18.4 (6.4)	18.1 (6.4)
Total fat (% of energy)	30.3 (10.7)	30.8 (11.4)	30.6 (11.4)	30.1 (10.5)	30.3 (11.5)
Saturated fat (% of energy)	10.1 (3.9)	10.3 (4.0)	10.3 (4.1)	10.0 (3.7)	10.2 (4.2)
Mono-unsaturated fat (% of energy)	11.6 (4.4)	11.8 (4.8)	11.8 (4.7)	11.5 (4.2)	11.6 (4.6)
Poly-unsaturated (% of energy)	5.8 (2.3)	5.9 (2.6)	5.8 (2.4)	5.9 (2.6)	5.9 (2.6)
Poly-unsaturated/saturated fat (ratio)	0.61 (0.20)	0.60 (0.20)	0.60 (0.19)	0.61 (0.24)	0.60 (0.21)
Total *trans* fat (% of energy)	1.39 (0.68)	1.47 (0.87)	1.44 (0.74)	1.42 (0.72)	1.45 (0.80)
Total n-3 fatty acids (% of energy)†	0.67 (0.33)	0.66 (0.33)	0.65 (0.45)	0.64 (0.33)	0.63 (0.30)
Fruit and vegetable intake (servings/day)	6.0 (2.6)	5.7 (2.4)	5.9 (2.5)	5.9 (2.6)	5.6 (2.7)
Vitamin D (IU/day)	366.4 (247.0)	364.1 (251.3)	352.6 (251.0)	350.8 (243.9)	341.8 (287.4)
Vitamin E(mg/day)	83.4 (175.8)	104.5 (211.7)	85.7 (190.3)	82.2 (175.9)	79.7 (172.9)
Caffeine (mg/day)	231.4 (205.2)	250.3 (220.1)	243.4 (224.1)	241.8 (215.1)	247.2 (216.4)

* Due to rounding, percentages may not sum to 100.0.

† Combined alpha-linolenic acid and long-chain omega-3 fatty acids (docosahexaenoic and eicosapentaenoic acids).

Higher phobic anxiety was associated with lower age-adjusted mean RTL *z*-scores (p-trend = 0.09); estimates were similar after adjustment for paternal age-at-birth, smoking, BMI and physical activity (p-trend = 0.15) (Table 2). However, the phobic anxiety-RTL association was characterized by a threshold effect (Table 3). Although women with CCI<6 had adjusted mean RTL *z*-scores of 0.02 standard units, those with CCI≥6 had mean RTLs of −0.09 standard units (mean difference = −0.10 standard units; p = 0.02 for difference in means). When compared to the adjusted mean RTL *z*-score for one year of age (−0.015 standard units, p<0.0001), the magnitude of this difference was equivalent to that observed for women 6 years apart in age.

**Table 2:** Least-squares mean leukocyte telomere length z-scores (standard errors), by phobic anxiety categories.

	*Crown-Crisp Index Phobic Anxiety Scores*					
	*0 or 1*	*2*	*3*	*4 or 5*	*≥6*	P-trend
***N***	1,782	1,024	816	1,022	599	
Age-adjusted	0.01 (0.02)	0.04 (0.03)	−0.01 (0.03)	0.02 (0.03)	−0.09 (0.04)	0.09
Multivariable-adjusted I*	0.01 (0.02)	0.04 (0.03)	−0.01 (0.03)	0.02 (0.03)	−0.09 (0.04)	0.15
Multivariable-adjusted II†	0.01 (0.02)	0.04 (0.03)	−0.01 (0.03)	0.02 (0.03)	−0.09 (0.04)	0.15

* Adjusted for age in years (continuous), paternal age-at-birth (15–24, 25–29, 30–34, 35–39, ≥40 years), pack-years of smoking (0, 0< to <20, 20 to <40, 40+), physical activity (continuous, MET-hrs/wk), body mass index (continuous, kg/m^2^).

† Adjusted for model I variables, plus chronic medical conditions/potential intermediates: hypertension (yes/no), dyslipidemia (yes/no), diabetes (yes/no), cardiovascular disease (yes/no).

**Table 3:** Least-squares mean leukocyte telomere length z-scores (standard errors), by high phobic anxiety.

	Crown-Crisp Index Phobic Anxiety Scores		
	*<6*	*≥6*	P-value‡
***N***	4,644	599	
Age-adjusted	0.02 (0.01)	−0.09 (0.04)	0.009
Multivariable-adjusted I*	0.02 (0.01)	−0.09 (0.04)	0.02
Multivariable-adjusted II†	0.02 (0.01)	−0.09 (0.04)	0.02

* Adjusted for age in years (continuous), paternal age-at-birth (15–24, 25–29, 30–34, 35–39, ≥40 years), pack-years of smoking (0, 0< to <20, 20 to <40, 40+), physical activity (continuous, MET-hrs/wk), body mass index (continuous, kg/m^2^).

† Adjusted for model I variables, plus chronic medical conditions/potential intermediates: hypertension (yes/no), dyslipidemia (yes/no), diabetes (yes/no), cardiovascular disease (yes/no).

‡ P-value for test of difference in least-squares means.

**Figure 1:**
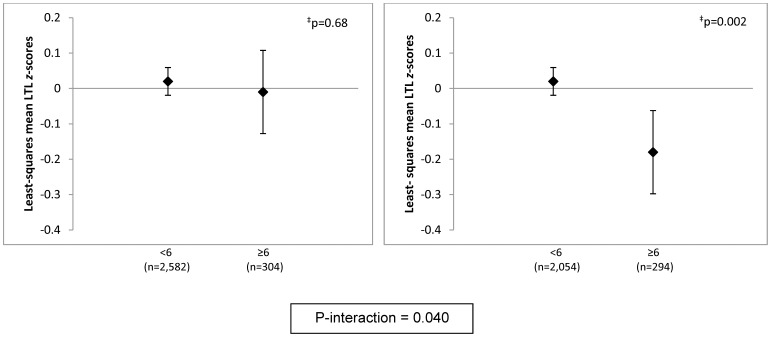
Adjusted* least-squares mean leukocyte telomere length *z*-scores, by phobic anxiety level, stratified by body mass index (<25 kg/m^2^ or ≥25 kg/m^2^). *, Models were adjusted for age in years (continuous), paternal age-at-birth (15–24, 25–29, 30–34, 35–39, ≥40 years), pack-years of smoking (0, >0 to <20, 20 to <40, ≥40), and physical activity (continuous, MET-hrs/wk). Least squares means and 95% confidence intervals are shown. N = 9 excluded due to missing body mass index. ^‡^, P-value for test of difference in least-squares means.

**Figure 2:**
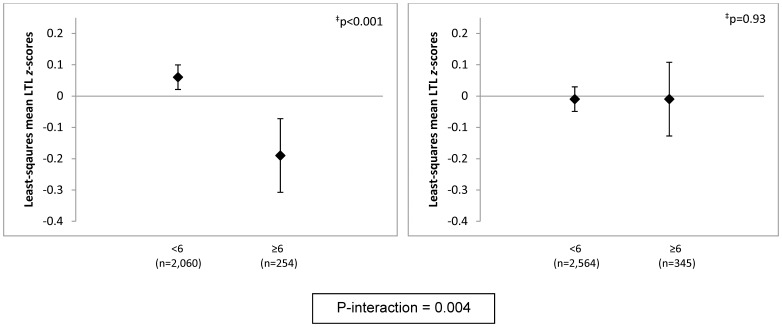
Adjusted* least-squares mean telomere length *z*-scores, by phobic anxiety level, stratified by smoking status (never or current/past). *, Models were adjusted for age in years (continuous), paternal age-at-birth (15–24, 25–29, 30–34, 35–39, ≥40 years), body mass index (continuous, kg/m^2^) and physical activity (continuous, MET-hrs/wk). Least squares means and 95% confidence intervals are shown. N = 20 excluded due to missing smoking status. ^‡^, P-value for test of difference in least-squares means.

**Figure 3:**
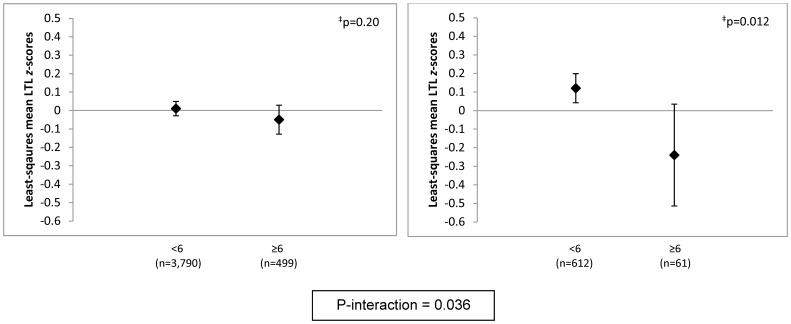
Adjusted* least-squares mean telomere length *z*-scores, by phobic anxiety level, stratified by paternal age (<40 or ≥40 years). *, Models were adjusted for age in years (continuous), pack-years of smoking (0, >0 to <20, 20 to <40, ≥40), body mass index (continuous, kg/m^2^) and physical activity (continuous, MET-hrs/wk). Least squares means and 95% confidence intervals are shown. N = 281 excluded due to missing paternal age. ^‡^, P-value for test of difference in least-squares means.

Further adjustment of the primary multivariate model for major medical conditions had no influence on results (Table 2). There were no interactions of high phobic anxiety with participant age, maternal age, physical activity or prior BZD use. However, significant interactions were observed by BMI, smoking and advanced paternal age (APA). Compared with leaner women (BMI<25 kg/m^2^), associations between high phobic anxiety and lower RTLs were stronger among overweight/obese women (Figure 1). Mean RTLs were lower overall among smokers, but there was no association between high phobic anxiety and lower RTLs; by contrast, the differences in telomere lengths were striking among never-smokers (Figure 2). The mean difference (95% CI) in RTL *z*-scores comparing never-smoking women with CCI≥6 vs. CCI<6 was −0.25 (−0.38,−0.12) standard units (p<0.001). There was no interaction between BMI and smoking or 3-way interaction of phobic anxiety, BMI and smoking. Finally, a significant phobic anxiety-paternal age interaction was identified; differences in RTLs were more pronounced among highly phobic women born to fathers aged ≥40 years (Figure 3). In the models excluding participants with prevalent CVD, diabetes or obstructive airways diseases, estimates were attenuated. For example, after exclusion of 330 women with CVD or diabetes, the mean difference in RTLs comparing women with CCI≥6 vs. CCI<6 was −0.09 standard units (p = 0.04); with exclusion of an additional 264 participants with obstructive airways diseases, the mean difference was −0.07 standard units (p = 0.12). Significant interactions with BMI, smoking and APA remained after all chronic disease exclusions, but estimates were attenuated: e.g., among never-smokers, the mean difference in RTLs comparing women with CCI≥6 vs. CCI<6 was −0.20 standard units (p = 0.004).

In exploratory analyses addressing individual CCI items, endorsement of the response option that indicated the highest possible symptom level for a given item was generally associated with lower age-adjusted mean RTLs (Table S1). However, estimates appeared strongest for women with highest endorsement of two items that map to panic and agoraphobia (i.e., compared to women who did not endorse them at all): “feel panicky in crowds” and “uneasy traveling on buses or trains”. Nevertheless, due to the small number of participants who endorsed the highest symptom level for each item, estimates were predictably unstable; only one item, related to agoraphobia (“dislike going out alone”), was statistically significant (p = 0.047) (Table S1).

## Discussion

In this study of 5,243 women, high phobic anxiety was significantly associated with lower leukocyte telomere lengths. To aid interpretation of findings [40], we can compare the mean difference in RTL z-scores associated with high phobic anxiety to the estimate for women aged one year apart: compared to those with <6 points on the Crown-Crisp, women with ≥6 points had adjusted RTL z-scores equivalent to 6 years of age. In addition, effect modification was identified: associations were stronger among women who were born to older fathers, were heavier or never smoked. Results were somewhat attenuated after exclusion of participants with chronic diseases that may act as intermediates and/or present with higher subjective anxiety and also may be associated with telomere shortening – suggesting that findings may be partly explained by influences of phobic anxiety on risk of development of serious chronic diseases [19]. Finally, exploratory work indicated that endorsements of higher phobic symptom levels on individual CCI items were generally related to lower RTLs; estimates appeared strongest for items reflecting panic and agoraphobia. Overall, this study provides a key addition – phobic anxiety – to an emerging literature that posits mental distress and disorders as risk factors for accelerated aging [13], [14], [16], [57].

This report is consistent with earlier studies involving well-characterized samples of patients with diagnosed psychiatric disorders and controls [14], [58], [59]. However, there have been few larger-scale (n>500) population-based studies that specifically addressed anxiety and telomeres. Kananen et al. [60] measured RTLs in a sample (aged 30–87 years) of 321 participants with anxiety diagnosed via structured clinical interviews and 653 controls. Cases included both those with diagnostic manual criteria-level symptoms and those with core features of anxiety who did not meet full criteria; the majority of cases, however, met full criteria for generalized anxiety disorder (GAD), panic disorder, agoraphobia, and social and other phobia. Although RTLs were similar among cases and controls overall, cases had significantly shorter RTLs than controls in the older half of the sample (48–87 years). In contrast, Surtees et al. [61] did not observe significant differences in RTLs among 4,441 women (aged 41–80 years) with vs. without either 12-month or lifetime GAD, as diagnosed by participant self-assessment forms. Discrepant findings may have resulted from differences in ascertainment methods of diagnoses (self-assessment forms vs. structured interviews by clinical raters) or in the samples (e.g., age, gender, clinical features). For example, the symptoms of disorders under study by Kananen et al. [60] (specifically, panic and phobic disorders) have high overlap with the symptoms captured by the CCI; Surtees and co-workers [61] considered only GAD. Overall, our finding that lower RTLs were observed among those with the highest phobic symptoms – i.e., potentially a “caseness” level – compared to those below that threshold, is consistent with work in clinical samples. Nevertheless, it is not known whether women in the highest phobic symptom category would necessarily meet criteria [62] for diagnosis of an anxiety disorder (particularly, panic or agoraphobia), although this appears plausible.

The findings regarding effect modification of anxiety-telomere relations are novel and require confirmation/replication. Obesity may worsen oxidative stress and inflammation [63], [64], and relations of high phobic anxiety and telomere length may be influenced by body mass or adiposity stores. The findings for smoking were counter-intuitive. CCI scores were higher among ever- vs. never-smokers (Wilcoxon rank sum p = 0.001), smoking may trigger oxidative stress/damage [65], and RTLs were lower overall among smokers in our sample; one might have expected stronger phobic-telomere associations among smokers. Explanations for this finding include chance as well as putative influences of nicotine on key inflammatory mediators [66], [67] – possibly providing protection against telomere shortening for highly phobic smokers that would be absent for equally phobic non-smokers; however, the latter possibility is purely speculative. Finally, findings regarding advanced paternal age were intriguing. APA is associated with longer telomeres [43], but this also allows for more DNA abnormalities to accumulate and greater DNA fragmentation [68]. Intriguingly, APA has recently been related to psychiatric disorders [69], [70] and greater externalizing (vs. internalizing) behaviors in offspring [71]; in our cohort, paternal age ≥40 y vs. <40 y was associated with lower prevalence of high phobic anxiety (12% vs. 9%, χ^2^_(df = 1)_ = 3.84, p = 0.05). Thus, while explanations remain unclear, one speculative possibility is that high phobic anxiety might exert an exaggerated impact on vulnerable DNA, with resulting faster telomere shortening, among women born to older fathers; alternatively, greater overall variability in TLs among offspring of older fathers may enhance ability to detect phobic anxiety-TL relations.

Although the literature is at an early stage, there is biologic plausibility to support relations of anxiety to shorter telomeres, particularly via oxidative stress and inflammation. For example, in a study [72] of 362 healthy adults, higher tension-anxiety symptom level was correlated with an oxidative DNA damage marker, 8-hydroxydeoxyguanosine. In a previous NHS investigation [18], elevated inflammatory markers (tumor necrosis factor-α receptor II, soluble E-selectin and soluble intercellular adhesion molecule) were observed among diabetic women with the highest phobic anxiety (those with CCI≥6 were grouped in the same category with those with CCI = 4 or 5) [18]. Similarly, higher scores on the Spielberger State-Trait Anxiety Inventory were significantly correlated with elevated C-reactive protein, interleukin-6 and fibrinogen levels in 853 middle-aged adults [73]. Nevertheless, despite high prevalence of anxiety [20], few studies have focused on its relations to oxidative stress and inflammatory biomarkers. Thus, additional investigation is necessary to delineate potential impacts of anxiety on aging via these mechanisms.

Strengths of the current study include: use of a validated phobic anxiety scale; a large, well-characterized sample; and consideration of numerous potential health, lifestyle and socio-demographic confounders. Also, high phobic symptoms may have been present among these participants for decades [23] – highlighting the particular value of relating this exposure to RTLs, which are markers of cumulative aging. Indeed, a connection between early-life exposure to adverse mental health environments and consequences of accelerated aging is an intriguing possibility only recently examined in the literature [61], [74], [75].

Potential limitations should be considered. First, the cross-sectional design precludes establishment of a temporal association between phobic anxiety and telomere length. For example, the possibility of bi-directional links has been raised by recent animal work implicating oxidative stress in development of anxiety [76]. Also, individuals with shorter telomeres may have chronic diseases that contribute to persistence, or even worsening, of phobic symptom levels; however, this possibility appears unlikely to explain results entirely because key findings remained, albeit attenuated, after excluding individuals diagnosed with major comorbidities prior to blood collection. Second, RTL was a single measure, preventing estimation of associations between phobic anxiety and telomere attrition rate. Third, we lacked specific data on anxiety onset, duration and/or treatments and, thus, were unable to incorporate such factors into analyses. Also, we did not concurrently measure depression (such assessments began in NHS in 1992); thus, we cannot exclude confounding by depression (e.g., comorbidity of depressive disorder among persons with anxiety disorders has been estimated at 45% [77]). However, a potentially more likely possibility is that current depression among persons with high phobic anxiety would represent an intermediate variable: phobic conditions typically have early-life onset – decades before median onset of depression [20]; indeed, the “temporal primacy” of anxiety disorders was previously summarized by Kessler [23], [78]. Nevertheless, although it seems unlikely that true confounding by depression would completely explain results, we cannot make conclusions regarding on the role of depression in this analysis. Fourth, as in any observational study, residual confounding is possible. Finally, generalizability is a potential concern; the Nurses were predominantly (95%) of white and European race/ethnicity. Because telomere dynamics may differ among African-Americans and Hispanics [79], [80], the magnitude of associations may not apply to women of other ethnicities or to men. Nevertheless, basic biologic relations between anxiety and telomere lengths are likely to hold in all humans.

In summary, high phobic anxiety may be associated with shorter leukocyte relative telomere lengths in middle-aged and older women. Identification of these novel associations invites further investigation in large-scale prospective studies, with detailed ascertainment of anxiety and other mental health variables and repeated measures of telomere length. Furthermore, because phobic anxiety is usually the temporally primary condition in comorbidity involving other mental disorders (e.g., depression, substance abuse) [23], early intervention not only may mitigate detrimental impacts on aging and but also could avert additional downstream consequences of accelerated telomere shortening due to secondary development of other mental disorders or serious chronic medical conditions.

## Supporting Information

Table S1 Adjusted* least-squares mean telomere length ***z*** -scores, according to individual items on the Crown-Crisp Index,* Models were adjusted for age in years (continuous). Total N varies due to missing responses for each item. P-values are from tests of differences in least-squares means, where the lowest symptom level/non-endorsement is the reference category.
(DOCX)
