# Supplementary material for: High Phobic Anxiety Is Related to Lower Leukocyte Telomere Length in Women
Source: PLoS One. 2012 Jul 11;7(7):e40516. doi: 10.1371/journal.pone.0040516 (PMC3394740; doi:10.1371/journal.pone.0040516)
Supplement: Table S1 — Adjusted* least-squares mean telomere length z -scores, according to individual items on the Crown-Crisp Index,* Models were adjusted for age in years (continuous). Total N varies due to missing responses for each item. P-values are from tests of differences in least-squares means, where the lowest symptom level/non-endorsement is the reference category. (DOCX) [file pone.0040516.s001.docx]

**Table S1. Least-squares mean telomere length *z*-scores, according to individual items on the Crown-Crisp Index***

|  | **N** | **Mean (standard error)** | **P-value** |
| --- | --- | --- | --- |
| **Do you have an unreasonable fear of being in enclosed spaces such as shops, lifts, etc?** |  |  |  |
| Never | 4351 | 0.01 (0.02) |  |
| Sometimes | 706 | -0.05 (0.04) | 0.09 |
| Often | 75 | 0.02 (0.11) | 0.93 |
| **Do you find yourself worrying about getting some incurable illness?** |  |  |  |
| Never | 2688 | 0.01 (0.02) |  |
| Sometimes | 2479 | 0.00 (0.02) | 0.85 |
| Often | 52 | -0.19 (0.14) | 0.15 |
| **Are you scared of heights?** |  |  |  |
| Not at all | 1794 | -0.00 (0.02) |  |
| Moderately | 2943 | 0.02 (0.02) | 0.47 |
| Very | 486 | -0.07 (0.04) | 0.15 |
| **Do you feel panicky in crowds?** |  |  |  |
| Not at all | 4165 | 0.02 (0.02) |  |
| Moderately | 1039 | -0.04 (0.03) | 0.08 |
| Very | 16 | -0.28 (0.25) | 0.23 |
| **Do you worry unduly when relatives are late in coming home?** |  |  |  |
| No | 3555 | 0.01 (0.02) |  |
| Yes | 1651 | -0.01 (0.02) | 0.48 |
| **Do you feel more relaxed indoors?** |  |  |  |
| Not particularly | 4569 | 0.01 (0.01) |  |
| Sometimes | 495 | -0.00 (0.04) | 0.85 |
| Definitely | 162 | -0.04 (0.08) | 0.60 |
| **Do you dislike going out alone?** |  |  |  |
| No | 4660 | 0.01 (0.01) |  |
| Yes | 555 | -0.08 (0.04) | <0.05 |
| **Do you feel uneasy traveling on buses or trains even if they are not crowded?** |  |  |  |
| Not at all | 4564 | 0.01 (0.01) |  |
| A little | 613 | -0.01 (0.04) | 0.58 |
| Definitely | 22 | -0.21 (0.21) | 0.31 |

* Models were adjusted for age in years (continuous). Total N varies due to missing responses for each item. P-values are from tests of differences in least-squares means, where the lowest symptom level/non-endorsement is the reference category.
